# The Pedicle

**Published:** 1891-04

**Authors:** 


THE PEDICLE.—Should future cases present similar opportunities, and further experiences confirm Dr. Walker's views, he has made a contribution to the technique of ovariotomy, of no small value. Read the paper on page 432 in this issue. Dr. Walker found no pedicle, but had to amputate through the portion of the tumor constricted by the ligature. Here then, was an unpleasant dilemma: what to do with the jagged surface so calculated to harbor septic matter? The doctor was equal to the occasion, and promptly covered the raw surface with a fold of peritoneum. The case illustrates the necessity for a surgeon to possess good common sense, as well as scientific knowledge.

